# Challenges and Opportunities on Lipid Metabolism Disorders Diagnosis and Therapy: Novel Insights and Future Perspective

**DOI:** 10.3390/metabo11090611

**Published:** 2021-09-09

**Authors:** Zlatko Fras, Borut Jug, Peter E. Penson, Manfredi Rizzo

**Affiliations:** 1 Centre for Preventive Cardiology, Department of Vascular Medicine, Division of Medicine, University Medical Centre Ljubljana, SI-1525 Ljubljana, Slovenia; zlatko.fras@kclj.si (Z.F.); borut.jug@kclj.si (B.J.); 2 Chair of Internal Medicine, Medical Faculty, University of Ljubljana, SI-1000 Ljubljana, Slovenia; 3 School of Pharmacy and Biomolecular Sciences, Liverpool John Moores University, Liverpool L3 3AF, UK; p.penson@ljmu.ac.uk; 4 Liverpool Centre for Cardiovascular Science, Liverpool L7 8TX, UK; 5 Department of Health Promotion, Mother and Child Care, Internal Medicine and Medical Specialties (Promise), University of Palermo, 90133 Palermo, Italy

Dyslipidemia has been globally recognized, for almost seven decades, as one of the most important risk factors for the development and complications of atherosclerotic cardiovascular disease (ASCVD) [1]. Clinical trials and large epidemiological studies have provided strong evidence for the magnitude of low-density lipoprotein cholesterol (LDL-C) lowering (primarily by statins), consistently predicting ASCVD risk reductions [1]. It has been recently emphasized that patients at high cardiovascular risk, such as those with type-2 diabetes, are mainly at risk of ASCVD [2]; therefore, the prevention and treatment of the development and progression of atherosclerosis is still a major public health issue that needs to be addressed properly.

However, several questions still remain to be answered in relation to the comprehensive, higher-quality management of raised LDL-C levels. These include more consistent use of the highest possible doses of potent statins; easier prescription of combination therapies; faster and better identification of patients with dyslipidemia who would benefit from alternative treatment strategies, such as PCSK9 inhibition; as well as issues relating to the diagnosis and effective management of statin intolerance and its subjective equivalents [3,4,5,6,7,8]. In addition, researchers have not yet fully addressed the questions of when to start and how aggressively to treat hypercholesterolemia in children, adolescents, and the elderly, as well as whether to respond to the risk of ASCVD with statins in very-high- and high-risk patients with normal/low LDL-C concentrations [1,9,10].

Despite the unequivocal efficiency of statins, it remains to be determined to what extent the residual ASCVD risk is related to dyslipidemia, especially in patients with low LDL-C. Furthermore, questions remain as to the choice of the best possible management of patients with combined hyperlipidemia, moderate hypertriglyceridemia or atherogenic dyslipidemia [11]. We face these challenges in clinical practice in most patients with metabolic syndrome and type-2 diabetes [12,13]. This is also linked to the fact that LDL particles are very heterogenous, with different physico-chemical composition, metabolic behavior, LDL-receptor affinity, susceptibility to oxidation and, ultimately, different atherogenic potential [14]. Small, dense LDL particles are responsible for the formation and progression of atherosclerotic plaques, and have been recognized as an independent risk factor for cardiovascular diseases, even independently of LDL concentrations [15].

There is indeed increasing evidence suggesting that the quality (e.g., small dense particles), and not only the quantity (e.g., plasma concentrations), of LDL is associated with cardiovascular risk [16]. Elevated levels of small, dense LDL particles are found in many categories of patients at high cardiovascular risk [17,18,19,20,21] and are the key factors for the early stages of atherosclerosis (subclinical) and endothelial dysfunction, which both amplify the risk of cardiovascular events [22]. Beyond statins [23], some novel anti-diabetic medications have shown favorable effects on the above mentioned patho-physiological alterations [24,25,26], and increasing evidence for effective cardiometabolic prevention are coming from incretin-based therapies [27]. Furthermore, the role of increased levels of lipoprotein (a) and the potential clinical efficiency of the much more effective lowering of this lipoprotein is an increasingly challenging topic [28]. Additional topics of interest include the role of emerging therapies for elevating concentrations of high-density lipoprotein cholesterol (HDL-C) and augmenting HDL particle functionality [29,30], as well as the modulation of lipoprotein-associated phospholipase A2 [31].

Although the available options for pharmacologic interventions are fortunately expanding, further well-designed studies are needed to assess the clinical effectiveness and long-term safety of any of these new pharmacological options. The majority of current preventive cardiology guidelines encompass numerous, increasingly evidence-supported recommendations on the way to approach the difficulties of poor compliance and adherence to long-term lipid-lowering therapy [2,12]. Furthermore, different aspects of the diagnosis and therapy of dyslipidemia are worthy of discussion in the era of contemporary medicine. In particular, one can highlight the so-called “4P” (predictive, preemptive, personalized, and participatory) approach, which primarily considers perspectives on the more efficient use of individual genetic information for both diagnostic and therapeutic purposes. In general, a more personalized but comprehensive approach is required instead of a “one-size-fits-all” intervention.

Furthermore, during the current pandemic, particularly careful management of cardiometabolic risk is necessary, since diabetes, obesity and hypertension are associated with an increased risk of the most severe forms of COVID-19, and its associated mortality [32]. In addition, cardiometabolic complications are increasing for many concomitant reasons, such as reduced physical activity, altered eating behaviors and a lack of access to healthcare facilities for non-COVID patients [33]. In this context, the importance of managing lipid alterations and the role of statins during the COVID-19 pandemic has been recently discussed [34,35]. In summary, with this Special Issue of Metabolites, it is our aim to critically review the available evidence on some of the above-listed challenges and opportunities. We wish to provide the medical and scientific community with the best update on current and novel lipid diagnostic tools, as well as on treatment options, in order to reduce the burden of ASCVD.
